# Supplementary material for: The Role of Elastic Stresses on Leaf Venation Morphogenesis
Source: PLoS Comput Biol. 2008 Apr 11;4(4):e1000055. doi: 10.1371/journal.pcbi.1000055 (PMC2275310; doi:10.1371/journal.pcbi.1000055)
Supplement: Text S1 — A minimal model with scale invariance properties. We present here a toy model that has the minimal hierarchical properties we expect to obtain in the full simulation. It may be useful to better appreciate the results of the full modeling. (0.16 MB PDF) [file pcbi.1000055.s001.pdf]

Supporting information

1 A minimal model with scale invariance properties

In view of the rather complicated technical characteristics of the full model we present in this paper, it may be appropriate to present here a toy model that has the minimal hierarchical properties we expect to obtain in the full simulation. As a price for its simplicity, this toy model produces patterns that are unrealistic in its global appearance. However, clarifying the hierarchical properties of this toy model may be important to better appreciate the results of the full modeling.

The toy model is defined as follows. Let us consider a rectangular surface. In the beginning, it is assumed that this surface has a very small area and represents the germ of the leaf that will grow. When its (linear) size reaches the critical value l_c, a new vein of unitary width appears, dividing the original surface in two. The system continues to grow isotropically, and every time a sector free of veins reaches a (horizontal or vertical) length l_c, a new vein is nucleated, dividing this sector in two. We consider that the new vein does not appear necessarily in the middle of the sector that is divided, but in an arbitrary position, with some probability distribution (most probably in the middle, and less toward the borders). This eliminates the existence of four veins junctions, which are rarely observed in real leaves. A few steps of this process are illustrated in Fig. 1. In this figure, all stages have been plotted as of the same size, i.e., we use the same 'zooming out' procedure as in the full

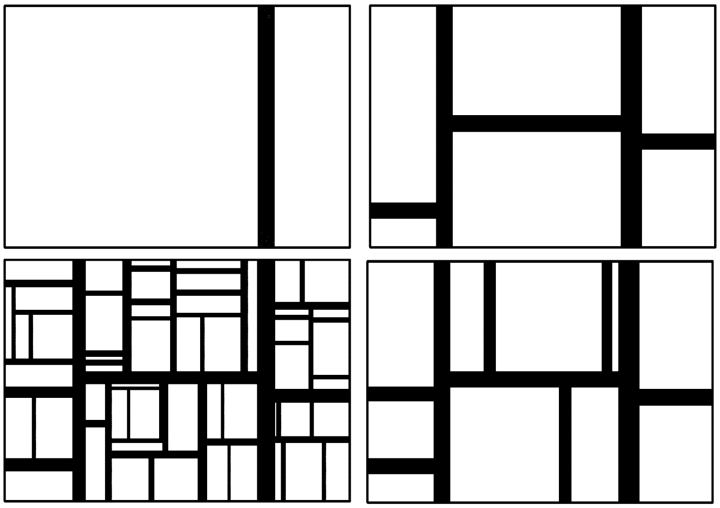

Fig. 1. Minimal model. Four steps in the division process.

model, and then Fig. 1 is the equivalent in the toy model of Figs. 3 and 4 of the paper.

This very simple model admits and equally simple calculation of the statistics of segments lengths and widths. In fact, first of all, it is easy to see that the typical length $L(w)$ of a segments of width w is independent of w, as segments get divided by thinner ones, independently of its width. This is true of course if the model is iterated infinitely. Otherwise we should get a cutoff at low w, with $L(w)$ going to zero for w going to zero.

Another interesting result is the scaling law of the number of segments $N(w)$ of a given width w. The total length of segments of width w is roughly $1/w$, as they appeared to divide a pattern with typical size $\sim l_c$ in pieces of smaller size. Since at the end the mean length of segments is independent of w, the number of segments of width w is $\sim w^{-1}$. However, in this estimation the implicit assumption is made that sectors are progressively divided in halves. If we want to go to a continuous description, this has to be taken into account,

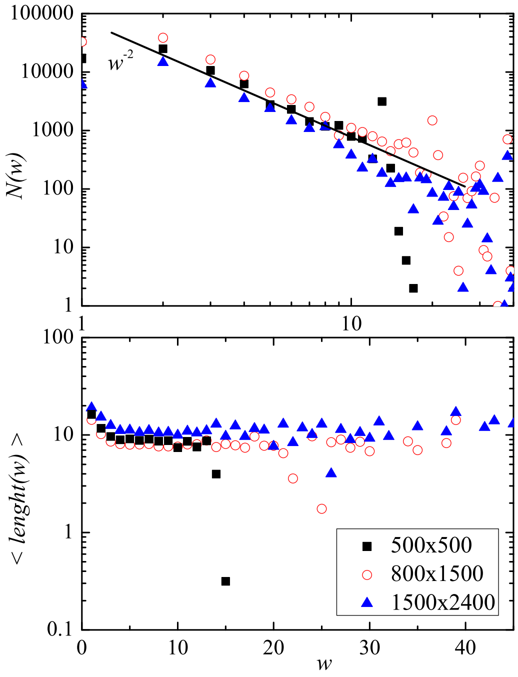

Fig. 2. Statistical behavior of the minimal model. Top panel: Histogram of the number of segments of a given width, $N(w)$ vs. w. Lower panel: Histogram of the average length of segments of width w. We show results for forty realizations in three system sizes.

and the result of creating a continuous histogram for $N(w)$ is that the number of segments of width w gets an additional w^{-1} factor, thus we obtain $N(w) \sim w^{-2}$.

The present simple model and its expected statistical behavior is a good benchmark to validate the numerical algorithms for segment location and counting we use in the full simulation. To do this we have run different configurations of the model and made the counting of segments length and width using the full machinery that has been explained in detail in Ref. (1). The results can be seen in Fig. 2. We confirm that in this toy model the mean length of segments is independent of its width, and the number of segments with a given width is

$N(w) \sim w^{-2}$. These results are consequence of the hierarchical way in which the patterns is constructed, and form the basis on which the results of the full simulations can be analyzed.

References

[1] Bohn, S., Andreotti, B., Douady, S., Munzinger, J., and Couder, Y. (2002). Phys. Rev. E **65**, 061914-061925.
